# Lipid Dysregulation Induced by Gasoline and Diesel Exhaust Exposure and the Interaction with Age

**DOI:** 10.3390/toxics12040303

**Published:** 2024-04-19

**Authors:** Yutong Gao, Xinzhuo Zhang, Xinting Li, Jinsheng Zhang, Zongyan Lv, Dongping Guo, Hongjun Mao, Ting Wang

**Affiliations:** 1 Tianjin Key Laboratory of Urban Transport Emission Research, State Environmental Protection Key Laboratory of Urban Ambient Air Particulate Matter Pollution Prevention and Control, College of Environmental Science and Engineering, Nankai University, Tianjin 300071, China; 2 Department of Visual Optics Medicine, Tianjin Medical University, Tianjin 300070, China

**Keywords:** gasoline exhaust, diesel exhaust, lipid metabolism, liver function, oxidative stress

## Abstract

Limited knowledge exists regarding gasoline and diesel exhaust effects on lipid metabolism. This study collected gasoline and diesel exhaust under actual driving conditions and conducted inhalation exposure on male young and middle-aged C57BL/6J mice for 4 h/day for 5 days to simulate commuting exposure intensity. Additionally, PM_2.5_ from actual roadways, representing gasoline and diesel vehicles, was generated for exposure to human umbilical vein endothelial cells (HUVECs) and normal liver cells (LO2) for 24, 48, and 72 h to further investigate exhaust particle toxicity. Results showed that diesel exhaust reduced total cholesterol and low-density lipoprotein cholesterol levels in young mice, indicating disrupted lipid metabolism. Aspartate aminotransferase and alanine aminotransferase levels increased by 53.7% and 21.7%, respectively, suggesting potential liver injury. Diesel exhaust exposure decreased superoxide dismutase and increased glutathione peroxidase levels. Cell viability decreased, and reactive oxygen species levels increased in HUVECs and LO2 following exposure to exhaust particles, with dose- and time-dependent effects. Diesel exhaust particles exhibited more severe inhibition of cell proliferation and oxidative damage compared to gasoline exhaust particles. These findings provide novel evidence of the risk of disrupted lipid metabolism due to gasoline and diesel exhaust, emphasizing the toxicity of diesel exhaust.

## 1. Introduction

Road traffic represents a major source of air pollutants in cities, exposing pedestrians and vehicle passengers to high concentrations of locally generated pollutants [1]. Various air pollutants are emitted from road vehicles, including but not limited to black carbon (BC), elemental carbon (EC), carbon monoxide (CO), hydrocarbons (HC), nitrogen oxides (NOx), nitrogen dioxide (NO_2_), fine particulate matter (PM_2.5_, aerodynamic diameter of ≤2.5 μm), etc., which contribute to the complexity of pollution compositions and concentrations of vehicle exhausts. Fuel type is one of the main influence factors of air pollutants derived from vehicle emission [2,3]. The most common fuel in private road passenger transport is gasoline, whereas diesel is the most common fuel in public road passenger and freight transit [4]. Most of the existing heavy-duty vehicles and some types of medium- and light-duty vehicles, including public buses, trucks, machinery, etc., are run with diesel [5]. Diesel vehicles emit a higher proportion of BC and NOx compared to gasoline vehicles [6]. The study in an exemplary emission hotspot of Germany’s North Rhine-Westphalia has shown that diesel vehicles contribute 71% of PM_10_ (of an aerodynamic diameter of ≤10 μm) emissions and 91% of NOx emissions, while gasoline vehicles contribute 29% of PM_10_ emissions and 9% of NOx emissions [7]. In China, it is reported that gasoline vehicles are responsible for 80.9% of CO, 77.6% of HC and 4.8% of NOx from total vehicle emissions, while 18.0% of CO, 11.4% of HC, 88.8% of NOx and >99% of PM are produced by diesel vehicles, according to the China Mobile Source Environmental Management Annual Report in 2021 (https://www.mee.gov.cn/hjzl/sthjzk/ydyhjgl/202109/t20210910_920787.shtml accessed on 1 August 2023).

Epidemiological studies have suggested the adverse health effects of diesel and gasoline exhaust exposure [8,9,10,11,12]. Recent experimental studies have mainly focused on the health impacts induced by diesel exhaust particles. In a study by Vesterdal et al. [13], in vivo and in vitro experiments using liver cells demonstrated oxidative stress and lipid accumulation following exposure to diesel exhaust particles. Through an in vitro cell study, Tseng et al. [14] found that inhalation of diesel exhaust particles induced oxidative stress in endothelial cells, leading to endothelial cell apoptosis. Lung exposure to diesel engine exhaust particles (DEP) has been associated with oxidative stress and lipid accumulation in the livers of obese diabetic mice [15]. In vivo studies by Miller et al. [16] demonstrated increased atherosclerotic plaque size and plasma lipid peroxidation in mice exposed to DEP. An in vitro cell study on gasoline exhaust particles showed that ultrafine particles present in gasoline exhaust also induced significant oxidative stress, lipid peroxidation, and cell inflammation [17]. In summary, the primary mechanism may involve the induction of inflammation and oxidative stress [18,19], with reactive oxygen species (ROS) generated by oxidative stress leading to the release of vascular permeability factor/vascular endothelial growth factor A, which affects the permeability of intercellular adhesion junctions [20]. Subsequently, particles may pass through the vascular endothelial-calcium-VE-cadherin network and enter the circulatory system [21], potentially affecting lipid metabolism in the human body. In actual situations, the penetration of gasoline and diesel fuels is diverse among on-road vehicle fleets in different regions. The different fuel-type compositions of vehicle fleets cause spatial variability in exhaust emissions, which may result in varying degrees of health effects [22]. In the downtown area, the vehicle fleet is mainly composed of gasoline vehicles, while there is a considerable proportion of diesel vehicles in the suburban area [23]. Therefore, in the real world, there are significant differences in the health effects of traffic-related air pollution on people living in downtown and suburban areas. However, there is still a lack of direct and comprehensive comparison of the health effects of gasoline and diesel emissions exposure under actual traffic exposure levels.

Lipid metabolism plays a critical role in the synthesis, breakdown, and utilization of fats in the human body, which is crucial for maintaining energy balance and storing body fat. Abnormal lipid metabolism is closely associated with metabolic diseases such as diabetes [24] and cardiovascular diseases, including coronary heart disease, myocardial infarction, and stroke [25], potentially leading to lipid deposition in arterial walls and the development of atherosclerosis. Lipid metabolism abnormalities may interact with inflammatory and oxidative stress conditions [25], forming a vicious cycle that accelerates disease progression. In addition, the lipid metabolism undergoes alteration with advancing age [26,27]. With the aging process, the cell’s protective ability degrades. The aging process induces the progressive degeneration of cell protective ability, leading to well-defined phenotypic changes in blood vessels and a heightened susceptibility to cardiovascular system diseases [28]. Moreover, young individuals are in a developmental stage characterized by hormonal fluctuations and rapid maturation of organ systems; their lipid metabolism systems are typically more sensitive, which implies that metabolic processes are vulnerable to environmental perturbations during puberty [29]. Existing evidence has proven that age is one of the most important factors in the health effects of environmental pollutants [30,31]. However, the comparative study of the impacts of exposure to diesel and gasoline exhaust on lipid metabolism remains unclear. Furthermore, study of the interaction effect between vehicle exhaust and age is still lacking. Therefore, young and middle-aged C57BL/6J mice were exposed to diesel and gasoline exhaust from vehicles on a chassis dynamometer under actual traffic exposure levels. The effects on blood lipids, liver function, and oxidative stress levels were investigated to evaluate the impacts of diesel and gasoline exhaust on lipid metabolism. Furthermore, based on the results of the in vivo experiment, in vitro experiments using human umbilical vein endothelial cells (HUVECs) and human normal liver cells (LO2) were conducted to investigate the potential mechanisms of diesel and gasoline exhaust exposure. This research will provide scientific evidence for understanding the link between diesel and gasoline exhaust exposure and metabolic diseases.

## 2. Materials and Methods

### 2.1. Mouse Exposure

#### 2.1.1. Experimental Animals

Young (8-week-old) and middle-aged (24-week-old) male C57BL/6J mice were purchased from the Laboratory Animal Center of the Academy of Military Medical Sciences (Beijing, China). After 1-week acclimation, all mice were exposed to gasoline/diesel vehicle exhaust by inhalation exposure. The young mice were divided into three groups: the gasoline exhaust (GE) group, the diesel exhaust (DE) group, and the control group (exposed to ambient air) (n = 6 in each group). Middle-aged mice were grouped in the same manner as young mice. To simulate the average intensity of personal exposure to traffic-related air pollution in daily commutes, inhalation exposure experiments were conducted for 4 h/day for 5 days in an exposure chamber. The mice were provided with water and food *ad libitum*. All procedures were approved by the Animal Experiments Ethical Committee of Nankai University and carried out in conformity with the Guide for Care and Use of Laboratory Animals.

#### 2.1.2. Exposure Process in Mice

Gasoline and diesel vehicle exhaust was emitted from the experimental vehicles on the chassis dynamometer (Figure S1A). During the 4 h exposure period, the experimental vehicle was operated under a 1 h New European Driving Cycle (NEDC), including acceleration, deceleration, constant speed, and idle speed modes; 1 h idle speed; 1 h 40 km/h constant speed; and 1 h 80 km/h speed modes to simulate the actual average commuting driving conditions. The collected exhaust was diluted eight times with ambient air and introduced into the exposure chamber at a rate of 15–20 L/min. The concentrations of pollutants in the exposure chamber were measured using scanning mobility particle sizers and aerodynamic particle sizers.

The exposure chamber was made of medical-grade stainless steel (316L MS) and had a volume of 40 L [500 (L) × 400 (W) × 200 (H) mm]. There were four multi-functional openings in the chamber, which were used for air intake, air outlet, and detection of concentrations of PM_2.5_ or gaseous pollutants. The exposure chamber could accommodate two standard independent ventilation cages for mice. The temperature was controlled at 20–25 °C during the exposure period (Figure S1). Twelve hours after the final exposure, mice were sacrificed.

The concentrations of PM_2.5_ in the exposure chamber were continuously measured by a scanning mobility particle sizer (SMPS, 3898) spectrometer (TSI Inc., Shoreview, MN, USA) and an aerodynamic particle sizer (APS, 3321) spectrometer (TSI Inc., Shoreview, MN, USA). The concentrations of gaseous pollutant emissions were measured by a SEMTECH-DS Portable Emission Measurement System (PEMS) (Sensors Inc., Saline, MI, USA). The concentrations of gaseous pollutants and the mass concentrations of PM_2.5_ in the chamber are shown in Table 1.

#### 2.1.3. Biochemical Analysis of Serum

Blood samples were collected from the retro-orbital veins of mice while they were under isoflurane anesthesia. To extract supernatant serum, blood samples were placed into 1.5 mL microcentrifuge tubes and centrifuged at 3000 rpm for 10 min. Serum samples (100 μL/sample) were transferred to a new tube and tested using an automated biochemical analyzer (Model 7020, Hitachi, Tokyo, Japan). Total cholesterol (TC), triglyceride (TG), high-density lipoprotein cholesterol (HDLc), low-density lipoprotein cholesterol (LDLc), alkaline phosphatase (ALP), alanine aminotransferase (ALT), and aspartate aminotransferase (AST) levels were analyzed.

#### 2.1.4. Determination of Oxidative Stress Indicators

The aortic and liver tissues of mice were pretreated to obtain supernatant, and the supernatant and serum were taken to measure the oxidative parameters. The tissue samples were stored in liquid nitrogen until assayed for superoxide dismutase (SOD), glutathione peroxidase (GSH-Px), and malondialdehyde (MDA). All parameters were measured using enzyme-linked immunosorbent assay (ELISA) kits as directed by the manufacturer (Beyotime Biotechnology Co. Ltd., Beijing, China).

### 2.2. In Vitro Experiment

#### 2.2.1. PM_2.5_ Collection

To investigate the in vivo toxicity of gasoline and diesel exhaust particles, PM_2.5_ samples were collected in two traffic micro-environments, including the Wujinglu Tunnel located in the urban area of Tianjin City and the typical collection and distribution port highway Teda Street located in Tianjin Port. According to our previous research [32], the Wujinglu Tunnel traffic is mainly composed of gasoline vehicles (with diesel vehicles accounting for only about 2%). As an important gathering and distribution channel for Tianjin Port, which has the eighth largest cargo volume in the world, Teda Street traffic is mainly composed of diesel vehicles transporting goods [33]. Therefore, the Wujinglu Tunnel and Teda Street can represent the actual traffic pollution exposure levels of gasoline and diesel vehicles as the main vehicle types, respectively. PM_2.5_ was sampled from 06:30 to 21:30 and 22:00 to 06:00 from 28 August to 7 September in 2021 in Wujinglu Tunnel and from 7:30 to 17:00 and 17:20 to 7:20 from 12 to 22 June 2021 on Teda Street, respectively. The sampling height was approximately 1.5 m above ground level, within the average human respiratory zone, and approximately 1.5 m away from road traffic. The quartz fiber filters (90 mm diameter, PALL) for PM_2.5_ collection were baked at 600 °C in a muffle furnace for 2 h and then equilibrated for up to 72 h at a constant temperature (22 ± 1 °C) and relative humidity (35 ± 1%). Air samples were collected at a speed of approximately 100 L/min using a medium-volume sampler (TH-150AII, Tianhong, Wuhan, China) with a PM_2.5_ size-selective inlet. The sampled filters were wrapped with annealed aluminum foil and stored in a refrigerator at −18 °C until analysis. Particle size distribution was measured using a Dekati^®^ Electrical Low-Pressure Impactor (Dekati^®^ ELPI+, Kangasala, Finland). The ELPI+ continuously monitored the number and concentration of particles. The typical quantity size distribution of PM_2.5_ from Wujinglu Tunnel and Teda Street is shown in Table S1.

The sampled filters were cut to sizes of 2 cm × 1 cm, which were then put in the sample bottle, and 100 mL of deionized distilled water was added to soak them. Extraction by ultrasonic sonication was performed for 2 h after soaking for 30 min. The liquid containing PM_2.5_ was filtered through six layers of sterile gauze and centrifuged at 12,000 rpm at 4 °C for 30 min. Detached PM_2.5_ was then vacuum-freeze dried, and the mass was weighed [34]. PM_2.5_ was resuspended in a certain amount of sterile saline to achieve PM_2.5_ suspensions and stored at −20 °C until further experimentation. During the sonication process, the container was covered to minimize the risk of contamination and loss of volatile components; further, the freeze-drying process was conducted under low temperature and reduced pressure conditions to directly sublime the water content from the solid state without passing through the liquid phase. This method helped to maximize the retention of volatile and water-soluble components in the sample, thereby reducing their loss and ensuring long-term stability. The preparation method for this PM_2.5_ particle suspension has been widely used in existing relevant studies and has been proven effective [35,36,37].

#### 2.2.2. Cell Culture

Human umbilical vein endothelial cells (HUVECs) and human normal liver cells (LO2) were obtained from ScienCell (Carlsbad, CA, USA) and cultured in a humidified incubator at 37 °C with 5% CO_2_. HUVECs were cultured in endothelial cell medium (EndoCM), while RPMI 1640 medium was used for LO2 cell culture. For all of the experiments, HUVECs between passages 7 and 9 were utilized. The cells were divided into three groups: (1) the control group (medium only); (2) gasoline exhaust particles (GE particles in medium); and (3) diesel exhaust particles (DE particles in medium). The human daily inhalation dose of PM_2.5_ was calculated based on actual PM_2.5_ measurements (~30 μg/m^3^), as described in our previous study [34]. According to the formula shown in Text S1, the daily inhaled mass of PM_2.5_ for humans was determined to be 345.9 μg. In the preliminary experiments, exposure to estimated human daily intake doses of PM_2.5_ caused severe inhibition of cell viability. Therefore, we conducted in vitro experiments using doses equivalent to 1/100 and 1/50 of the estimated human daily intake of PM_2.5_. Each well was cultured with 200 μL of cell culture medium containing HUVECs and LO2 cells. Thus, the doses administered in vitro were 17.3 μg/mL and 34.6 μg/mL, respectively.

#### 2.2.3. CCK-8 Assay for Cell Viability

Cell viability was assessed using Cell Counting Kit-8 (CCK-8, Beyotime Biotechnology Co., Ltd., Shanghai, China) assays. HUVECs and LO2 cells in the logarithmic growth phase were seeded in 48-well plates at a density of 4 × 10^3^ cells per well. Each well was supplemented with 200 μL of medium containing the corresponding pollutants, and the medium was changed every 24 h during the incubation period. After culturing for 24, 48, and 72 h under the experimental conditions, the cells were incubated with CCK-8 reagent (10 μL/well) for 4 h [38]. Following the kit instructions, CCK-8 optical density (OD) absorbance at 450 nm was measured to judge the effect of exhaust particulate matter on cell viability.

#### 2.2.4. Reactive Oxygen Species (ROS) Levels

HUVECs and LO2 cells in the logarithmic growth phase were seeded in 6-well plates at a density of 5 × 10^6^ cells per well. Each well was supplemented with 1 mL of medium containing the corresponding pollutants, and the medium was changed every 24 h during the incubation period. After culturing for 24, 48, and 72 h, 10 μM DCFH-DA (Sigma-Aldrich, Saint Louis, MO, USA) was added to each well and incubated at 37 °C for 45 min. Subsequently, the average fluorescence intensity was measured using a FACS Calibur flow cytometer (BD, San Jose, CA, USA) after collection to assess the impact of exhaust particles on cellular ROS levels.

### 2.3. Statistical Analysis

GraphPad Prism Software Version 8.3.4 (San Diego, CA, USA) was used for the statistical analysis. A student’s *t*-test and one-way analysis of variance (ANOVA) with a subsequent Bonferroni’s multiple comparisons test were performed for the statistical significance test. A P-value of less than 0.05 was considered statistically significant. Experimental data were presented as the mean and standard error of the mean.

## 3. Results

### 3.1. Effect on Biochemical Parameters in Mice

**Figure 1:**
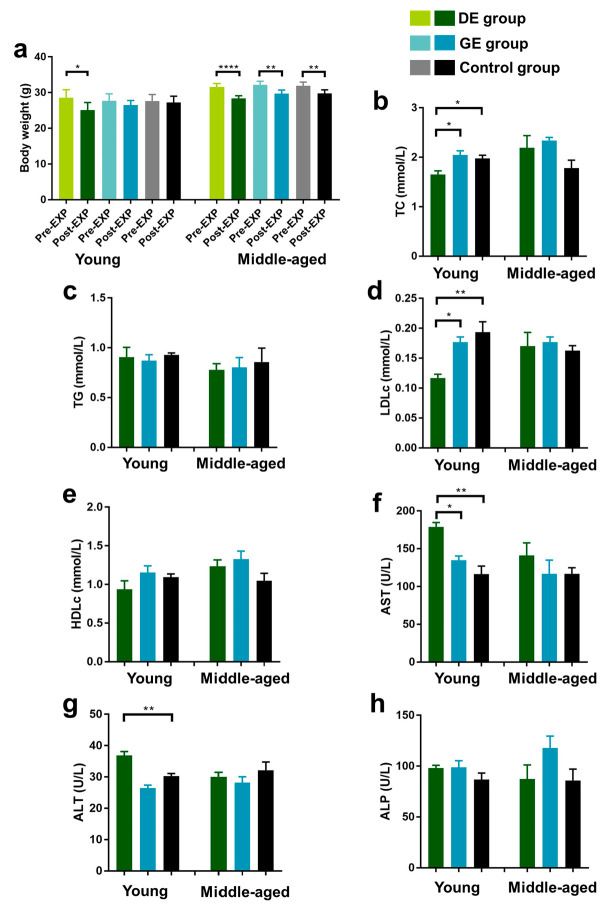
Effect of gasoline and diesel exhaust exposure on lipid profiles and liver function in C57BL/6 mice. (**a**) Body weight; (**b**) Serum total cholesterol level (TC); (**c**) Serum triglyceride level (TG); (**d**) Serum LDL-cholesterol level (LDLc); (**e**) Serum HDL-cholesterol level (HDLc); (**f**) Aspartate aminotransferase (AST); (**g**) Alanine aminotransferase (ALT); (**h**) Alkaline phosphatase (ALP) (Control group: exposure to ambient air; GE group: exposure to gasoline exhaust; DE group: exposure to diesel exhaust) (n = 6 in each group) (* *p* < 0.05; ** *p* < 0.01; **** *p* < 0.0001).

As shown in Figure 1a, exposure to diesel exhaust significantly reduced the body weight of young mice (*p* < 0.05), while the body weight of middle-aged mice was significantly reduced in all of the groups.

Serum samples were obtained to evaluate the effects of exhaust particles on lipid metabolism and liver function in mice, and TC, TG, LDLc, HDLc, AST, ALT, and ALP were determined. Blood lipid levels in the mice are shown in Figure 1b–e. For young mice, the TC and LDLc levels decreased significantly in the DE group (*p* < 0.01) compared with the control group, while there were no significant differences in all the lipid indicators between the GE and control groups. For middle-aged mice, there were no significant differences in TC, TG, LDLc, and HDLc level variations between the DE/GE and control groups.

The AST, ALT, and ALP levels are shown in Figure 1f–h. For young mice, diesel exhaust exposure elevated the AST and ALT levels by 53.7% and 21.7% (*p* < 0.01), respectively, compared to control group. Furthermore, compared to the GE group, the AST levels increased significantly in the DE group (*p* < 0.05). However, there were no significant differences in AST, ALT, and ALP level variations between the GE and control groups. For middle-aged mice, there were no significant differences in all of the liver function indicators between the DE/GE and control groups.

### 3.2. Effect on Oxidative Stress in Mice

Considering the negative effects of gasoline and diesel vehicle exhaust on the lipid mechanisms and liver function of mice, the oxidative stress of vehicle exhaust exposure on serum, aorta, and liver tissues was investigated. The SOD, GSH-Px, and MDA levels were measured.

#### 3.2.1. Serum

**Figure 2:**
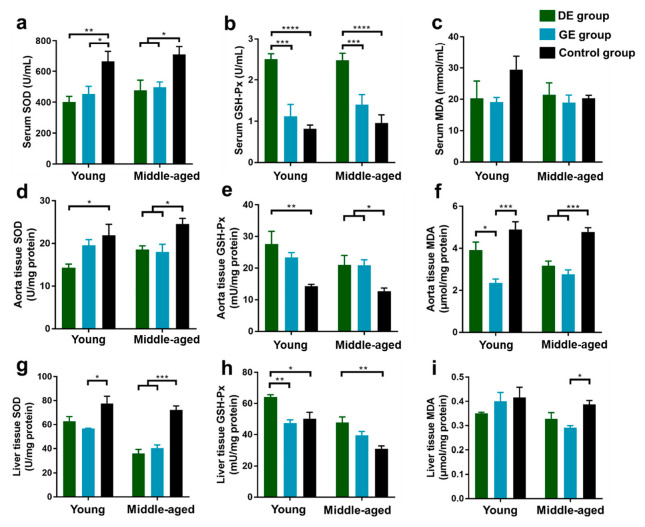
Effect of gasoline and diesel exhaust exposure on oxidative stress in C57BL/6 mice. (**a**) Serum superoxide dismutase (SOD); (**b**) Serum glutathione peroxidase (GSH-Px); (**c**) Serum malondialdehyde (MDA); (**d**) Aorta SOD; (**e**) Aorta GSH-Px; (**f**) Aorta MDA; (**g**) Liver SOD; (**h**) Liver GSH-Px; (**i**) Liver MDA. (Control group: exposure to ambient air; GE group: exposure to gasoline exhaust; DE group: exposure to diesel exhaust) (n = 6 in each group) (* *p* < 0.05; ** *p* < 0.01; *** *p* < 0.001; **** *p* < 0.0001).

As shown in Figure 2a,b, for young mice, the SOD activity in serum decreased significantly after exposure to both gasoline (GE group) and diesel exhaust (DE group) in comparison with the control group, while the GSH-Px activity increased significantly in the DE group. Moreover, there was a significant increase in GSH-Px levels in the DE group compared to the GE group. For middle-aged mice, the SOD levels decreased, and the GSH-Px activity in serum increased significantly in the DE group compared to the control and GE groups. Moreover, all of the effects of diesel exhaust exposure on SOD and GSH-Px activities were more pronounced than those caused by gasoline exhaust exposure. There were no significant differences in MDA levels among all of the groups of young and middle-aged mice (Figure 2c).

#### 3.2.2. Aorta

The SOD, GSH-Px, and MDA levels in aorta tissues after exposure to gasoline and diesel exhaust are shown in Figure 2d–f. For young mice, compared to the control group, the SOD level decreased, and the GSH-Px level increased significantly in the DE group, while the MDA level significantly decreased in the GE group. In addition, there was a more significant decrease in MDA levels in the GE group than in the DE group. For middle-aged mice, the SOD and MDA levels decreased, and the GSH-Px level increased significantly in both the GE and DE groups compared to the control group.

#### 3.2.3. Liver

The SOD, GSH-Px, and MDA levels in liver tissues after exposure to gasoline and diesel exhaust are shown in Figure 2g–i. For young mice, compared to the control group, the SOD activity decreased significantly in the GE group, and the GSH-Px activity increased significantly in the DE group. Furthermore, the increase in GSH-Px level in the DE group was more significant than that in the GE group. For the middle-aged group, the SOD levels decreased significantly in both the GE and DE groups compared to the control group, while the GSH-Px level increased significantly in the DE group, and the MDA level decreased significantly in the GE group.

### 3.3. Effect on Cell Viability of HUVECs and LO2

Based on the results of in vivo experiments concerning the effect on vascular and hepatic function, HUVECs and LO2 cells were incubated to explore the impacts and potential mechanisms of gasoline and diesel exhaust exposure. In addition, as shown in Table 1, the gaseous pollutants (CO, NO, and NO_2_) were below the Chinese National Air Quality Standard Grade II. However, the PM_2.5_ concentrations in the chamber during gasoline and diesel vehicle exhaust exposure periods were 50 and 370 μg/m^3^, respectively, which exceeded 0.43 and 9.57 times the standard. Therefore, PM_2.5_ samples emitted from gasoline and diesel vehicles were collected to conduct the in vitro exposure experiment.

**Figure 3:**
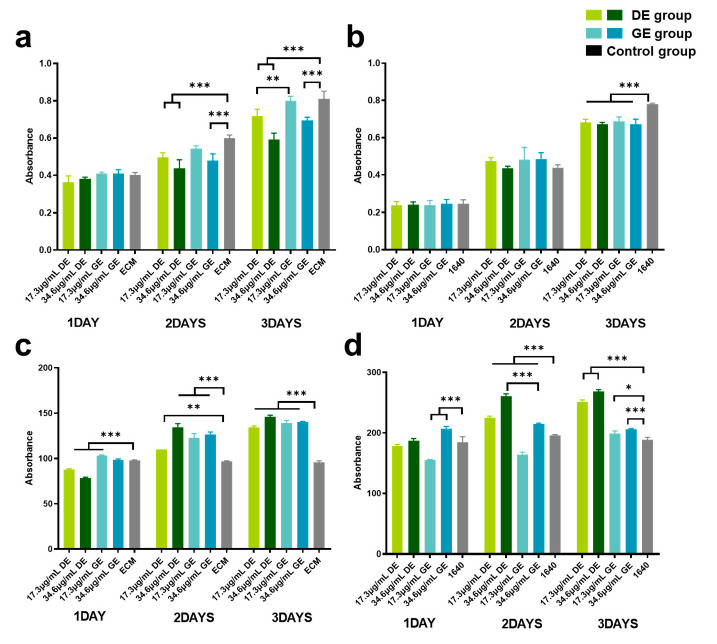
Effect of gasoline and diesel exhaust particle exposure on cell viability and ROS levels. (**a**) human umbilical vein endothelial cells (HUVECs) Cell Viability; (**b**) normal liver cells (LO2) Cell Viability; (**c**) HUVEC ROS Levels; (**d**) LO2 ROS Levels. (Control group: cells cultured in the respective medium only; Gasoline group: cells co-cultured with GE particles in the respective medium; Diesel group: cells co-cultured with DE particles in the respective medium.) (* *p* < 0.05; ** *p* < 0.01; *** *p* < 0.001).

Figure 3a,b illustrates the effects of gasoline and diesel exhaust PM_2.5_ on the viability of HUVECs and LO2 cells. As shown in Figure 3a, both gasoline and diesel exhaust particles significantly reduced HUVEC viability at 72 h (*p* < 0.001), with dose–effect dependence. In addition, diesel exhaust particles exhibited a more pronounced inhibitory effect on cell proliferation of HUVECs (*p* < 0.01) than gasoline exhaust particles. As depicted in Figure 3b, the cell viability of LO2 decreased significantly in both the GE and DE groups at 72 h (*p* < 0.001), but no significant difference was observed in the inhibitory effect on liver cell proliferation between the groups of different particle types and concentrations.

### 3.4. Effect on ROS Level

Figure 3c,d illustrates the impacts of gasoline and diesel exhaust particles on the oxidative stress levels in HUVECs and LO2. Significant increments in ROS levels in HUVECs and LO2 cells (*p* < 0.01) were observed. Moreover, the diesel exhaust particles exhibited more severe oxidation stress on liver cells than gasoline exhaust particles (*p* < 0.001) (Figure 3d).

## 4. Discussion

Vehicular emissions have been recognized as one of the most important sources of air pollutants [39,40]. The fuel type is the most important influence factor for air pollutants derived from vehicle emissions [2]. Gasoline- and diesel-fueled engines are the major constituent parts of on-road vehicles. Vehicular exhaust can rapidly enter the systemic circulation upon inhalation, induce systemic pathologies, affect lipid metabolism, and lead to various diseases [41,42,43,44,45]. Moreover, gasoline and diesel vehicle exhaust have great health impacts on people living in downtown and suburban areas, respectively. Therefore, to investigate the health impact of exposure to gasoline and diesel vehicle exhaust, in vivo experiments in C57BL/6J mice and in vitro experiments using human umbilical vein endothelial cells (HUVECs) and human normal liver cells (LO2) were conducted. In addition, the interaction effects between age and vehicle exhaust were evaluated in this study.

Regarding the analysis of blood lipids in mice, exposure to diesel exhaust led to a significant reduction in TC and LDLc levels in young mice. However, for middle-aged mice, there were no significant differences in all of the lipid indicators between the DE/GE and control groups. Previous studies have indicated a slight decrease in serum cholesterol after sub-chronic exposure to diesel exhaust in 10–12-week-old rats [46], with no significant impact observed in short-term exposures [47]. Furthermore, regarding the effects of PM_2.5_ on blood lipids, a group study conducted in Wuhan, China, targeting healthy young adults aged 18–30, revealed that for every 10 µg/m³ increase in PM_2.5_ concentration, the changes in TC and LDLc were −0.33% (95% *CI*: −0.64%, −0.01%) and −0.94% (95% *CI*: −1.53%, −0.35%), respectively [48]. Cholesterol is the sole precursor to all steroid hormones, like glucocorticoids responsible for blood sugar regulation and mineral corticoids required to regulate mineral balance and blood pressure [49,50]. In addition, cholesterol serves as a precursor for the biosynthesis of bile acids and vitamin D [49]. TC level is related to the regulation of stress responses, immune responses, electrolyte homeostasis, and the maintenance of secondary sexual characteristics, skeletal development, and bone homeostasis [51,52,53]. Furthermore, cholesterol can be obtained from circulating LDLc [54], and a decrease in LDLc levels is related to a low TC level. Lower TC and LDLc levels may impact cellular functions and hinder nutrient absorption and utilization. Therefore, exposure to diesel exhaust may have adverse effects on the growth and development of adolescents.

Regarding the analysis of liver function in mice, exposure to diesel exhaust significantly increased AST and ALT levels in young mice, while the effects of gasoline exhaust were not significant. Meanwhile, there was no significant influence of both gasoline and diesel vehicle exhaust exposure on all of the liver function indicators in middle-aged mice. The AST and ALT reflect the extent of hepatocellular injury [55]. Increments in serum AST and ALT were found in people with hepatosis compared to fit people [56]. The decrease in TC and LDL-C in serum is related to the liver, where liver cells are the site of cholesterol synthesis, and LDL-C transports cholesterol from the liver to other tissues. The decrement in TC and LDLc, as well as the increment in AST and ALT, are clinical diagnostic indicators of liver cell damage, suggesting possible acute injury to the liver [57,58]. Liver cells play a significant role in cholesterol synthesis, and LDLc transports cholesterol from the liver to other tissues. The liver is a central hub for balancing cholesterol from all sources and plays a pivotal role in modulating plasma LDLc [59]. It is possible that exposure to diesel exhaust may have an impact on the liver. Low LDLc levels could be caused by abnormalities in liver function or thyroid function [60,61]. Moreover, a study conducted by J. A. Bond et al. [62] suggested that the liver might serve as the primary site for the metabolism of carcinogens, such as 1-nitropyrene (1-NP), carried by diesel exhaust. Consistent with our findings, a cohort study conducted by Lin Xu et al. [63] on young adult males around the age of 35 found that exposure to diesel engine exhaust particles led to a significant increase in ALT and AST levels in participants, thereby increasing the risk of liver damage. Another study investigating the impact of nanoparticle rich-diesel exhaust (NR-DE) on the livers of 8-week-old male F344 rats revealed that NR-DE exposure led to increased AST and ALT activity in rats, and high concentrations of NR-DE further activated hepatic inflammatory signaling [64].

Therefore, exposure to vehicle exhaust, especially diesel vehicle exhaust, had a greater impact on young mice than on middle-aged mice in terms of blood lipid levels and liver function. Previous studies have classified the age of mice to correspond to different stages in the human lifecycle, where mice aged 6–8 weeks correspond to human adolescence and mice aged 24–26 weeks have fully matured in all aspects and have transitioned from adulthood to middle age [65]. Consistent with our findings, Ali K. Hamade et al. [66] observed that combined exposure to ozone and carbon black led to a decrease in heart rate and an increase in heart rate variability in mice, with these effects being more pronounced in young mice and relatively weaker in older mice. This difference in response may be attributed to the fact that the immune system of young mice has not fully developed, making them more sensitive to environmental pollutants and having a higher permeability of the respiratory epithelium to these pollutants [67,68]. Furthermore, both young and middle-aged mice were exposed to the same concentration of exhaust during the experiment, but due to the lighter body weight of young mice, their relative exposure to pollutants was greater, possibly resulting in more significant biological effects. Research by P.U. Simioni et al. [69] demonstrated that the aging process affects humoral and cellular-mediated immune responses, rendering older mice less sensitive to environmental challenges.

In order to further investigate the impact of gasoline and diesel exhaust on oxidative stress in mice, we measured oxidative stress markers, including SOD, GSH-Px, and MDA, in serum, aorta tissues, and liver tissues. Oxidative stress can be defined as an increase over physiological values in the steady-state concentrations of ROS. Antioxidant enzymes, such as SOD and GSH-Px, are identified as the first line of defense against the production and/or accumulation of ROS [70]. The SOD is a scavenger of endogenous ROS. Reduced SOD activity not only leads to inadequate removal of oxygen radicals but also induces MDA synthesis, resulting in protein and cellular damage and apoptosis [71,72]. GSH-Px is an essential enzyme for peroxide degradation, protecting the structural and functional integrity of cell membranes against peroxide damage [73]. MDA is a product of cellular membrane lipid peroxidation [74], which is commonly known as a marker of oxidative stress. Our results demonstrated that after exposure to gasoline and diesel emissions, SOD levels in the serum of young mice decreased significantly, and SOD levels in the aortic tissues and liver of adult mice also decreased significantly, with a greater impact observed from diesel exhaust. Both young and middle-aged mice showed significant increments in GSH-Px levels after diesel exhaust exposure and significant reductions in MDA levels in the aorta tissues after gasoline exhaust exposure. In existing studies, after instilling diesel exhaust particles into the trachea of mice, the ROS levels in the lungs first rose to a peak and began to decrease after 24 h [75]. Xu et al. [76] administered 50 μL of PM_2.5_ suspension (7.8 μg/g) via nasal drip to 8-week-old C57BL/6 mice. After three consecutive weeks of exposure, the total SOD level in the lung tissue of the mice decreased, and the GSH-Px level decreased after nine consecutive weeks of exposure. It can be inferred that short-term exhaust exposure leads to transient oxidative stress in the body, and the decrease in SOD levels and the increase in GSH-Px levels indicate that the body is recovering from tissue and organ damage. The rapid increase in GSH-Px activity in mice under stress led to a decrease in MDA levels. Overall, these results suggest that the degree of lipid oxidative damage caused by short-term acute exposure is relatively mild. This may be attributed to the timely activation of the endogenous antioxidant defense system, which effectively clears harmful oxidative substances and prevents their accumulation within the body, thereby mitigating severe oxidative damage. Young mice exhibited greater sensitivity to acute stimulation with a more pronounced oxidative stress response compared to middle-aged mice, which is consistent with the results observed for blood lipids and liver function.

These findings suggest that exposure to gasoline and diesel exhaust induces oxidative stress in the body, with diesel exhaust having a greater impact on oxidative stress compared to gasoline exhaust. A study by Fen Yin et al. [77] similarly reported increased levels of 9- and 13-hydroxyoctadecadienoic levels in the livers of mice exposed to diesel exhaust, indicating increased oxidative stress. Previous research has demonstrated that diesel exhaust emissions are more toxic than gasoline exhaust emissions when considering engines with equal horsepower [78]. Diesel vehicle emissions contain 2 to 40 times more particulate matter and 20 to 30 times more nitro-polycyclic aromatic hydrocarbons compared to gasoline vehicle emissions [79]. As shown in Table 1, in this study, the gaseous pollutants (CO, NO, and NO_2_) in the chamber during gasoline and diesel vehicle exhaust exposure periods were below the Chinese National Air Quality Standard Grade II. However, the measured concentrations of PM_2.5_ were 50 and 370 μg/m^3^, respectively, which exceeded 0.43 and 9.57 times the standard. Therefore, PM_2.5_ was assumed to be the major constituent in diesel and gasoline exhaust. In addition, based on the negative effects of gasoline and diesel exhaust exposure on vascular and liver function in in vivo experiments, HUVECs and LO2 cells were cultured in in vitro experiments to further explore the effects and potential mechanisms of PM_2.5_ in gasoline and diesel exhaust.

The results of the in vitro experiments demonstrated that exposure to diesel and gasoline vehicle exhaust particles resulted in decreased cell viability and increased ROS levels in both cell types with dose and time dependence, leading to inhibited cell proliferation, cellular oxidative damage, and impaired cellular functions. These findings are consistent with the current research results, suggesting that internalization of diesel engine exhaust particles into the cytoplasm of cells can induce oxidative stress [80], promoting cell apoptosis. Wang et al. [81] demonstrated that diesel engine exhaust particles induce oxidative stress and autophagy in endothelial cells, with cells attempting to eliminate the particles and generate free radicals after 2 h, followed by cell senescence and apoptosis after 12 h.

Furthermore, compared to gasoline vehicle exhaust particles, diesel vehicle exhaust particles exhibited more severe inhibition of HUVEC viability but higher oxidative stress in LO2 cells. HUVECs play a crucial role in forming the vascular barrier of blood vessels, preventing the entry of cells and large molecules from the blood into surrounding tissues, and regulating the exchange of fluids, gases, and solutes. The reduced cell viability of HUVECs caused by diesel exhaust particles may alter the permeability of blood vessel walls, leading to the leakage of blood components and extracellular fluid into surrounding tissues. This condition could increase intracellular oxidative stress, potentially initiating lipid oxidation reactions and lipid peroxidation damage, resulting in edema and inflammatory responses. Liver cells are one of the main sites for lipid breakdown and are central to cholesterol synthesis and metabolism. The reduced cell viability of liver cells induced by diesel exhaust particle exposure may slow down lipid breakdown, disturb cholesterol metabolism, and lead to the accumulation of fat in the liver, causing fatty liver and impacting lipid metabolism [77]. Grace V. Aquino et al. [82] found that ultrafine particles (UFPs) from diesel vehicle engine exposure caused concentration-dependent increases in ROS production in rat blood–brain barrier endothelial cells and perivascular microglia. Li et al. [83] demonstrated that UFPs affect signaling pathways in vascular endothelial cells and induce vascular endothelial oxidative stress via JNK activation.

However, this present study has limitations. The primary focus was to investigate the effects of gasoline and diesel exhaust emissions on lipid metabolism through biochemical and cellular experiments, without evaluating histopathological changes. Moreover, in our study, vehicle exhaust exposure induced oxidative damage in vivo and in vitro. Therefore, it is likely that inflammatory events could be caused. In future research, we will further incorporate pathological analysis and inflammatory indicator analysis.

In summary, combined with the in vitro and in vivo results, compared to gasoline vehicle exhaust, exposure to diesel vehicle exhaust had more substantial negative impacts on lipid metabolism and liver function via oxidative stress in live cells and cell injury in vascular cells. Moreover, the diesel exhaust exposure induced lipid metabolism dysfunction and acute liver injury in young mice, which revealed that puberty exhibited more instability and irregular regulation ability and more sensitivity to traffic emissions.

## 5. Conclusions

This study conducted in vivo and in vitro toxicological experiments on exposure to gasoline and diesel vehicle exhaust. The aim was to explore the impact of actual traffic exposure levels of gasoline and diesel vehicle exhaust on lipid metabolism. The results revealed that diesel exhaust exposure significantly decreased serum levels of TC and LDLc in young mice, suggesting potential disruptions in lipid homeostasis. Furthermore, it significantly elevated the levels of AST and ALT in young mice, indicative of potential acute liver injury. These effects were more pronounced in young mice, emphasizing the heightened susceptibility of adolescents to adverse impacts from vehicle emissions. Additionally, both gasoline and diesel exhaust exposure were found to decrease the viability of endothelial cells and liver cells, accompanied by elevated levels of ROS, implying cellular oxidative damage. Strikingly, the effects of exposure to diesel exhaust were more pronounced, indicating that diesel exhaust possesses greater toxicity, potentially exerting a more substantial influence on lipid metabolism and oxidative stress in the body compared to gasoline exhaust. Overall, our research provides evidence of the complex relationship between vehicle emissions from gasoline and diesel engines in actual traffic environments and lipid metabolism, oxidative stress, and age-related vulnerabilities. These findings are of paramount importance for mitigating health risks associated with vehicle emissions.

## Figures and Tables

**Table 1:** Concentrations of gaseous pollutants and the mass concentrations of PM_2.5_ in the chamber during the exposure period.

	PM_2.5_ (μg/m^3^)	CO (μg/m^3^)	NO (μg/m^3^)	NO_2_ (μg/m^3^)
GE group	50	25	2	-
DE group	370	50	20	4
Control group	6	-	-	-

## Data Availability

Data are contained within the article.

## Supplementary Materials

The following supporting information can be downloaded at: https://www.mdpi.com/article/10.3390/toxics12040303/s1, Figure S1: (A) The exposure chamber; (B) The exposure process; Table S1: Typical quantity-size distribution of PM2.5.

## Abbreviations

BC	black carbon;
EC	elemental carbon;
CO	carbon monoxide;
HC	hydrocarbons;
NOx	nitrogen oxides;
NO_2_	nitrogen dioxide;
PM	Particulate Matter;
OR	Odds Ratio;
DEP	diesel engine exhaust particles;
ROS	reactive oxygen species;
HUVECs	human umbilical vein endothelial cells;
LO2	human normal liver cells;
GE	gasoline exhaust;
DE	diesel exhaust;
NEDC	New European Driving Cycle;
SMPS	Scanning Mobility Particle Sizer;
APS	Aerodynamic Panicle Sizer;
TC	Total Cholesterol;
TG	Total Triglycerides;
HDLc	High-Density Lipoprotein Cholesterol;
LDLc	Low-Density Lipoprotein Cholesterol;
ALP	alkaline phosphatase;
ALT	alanine aminotransferase;
AST	aspartate aminotransferase;
SOD	Superoxide Dismutase;
MDA	Malondialdehyde;
GSH-Px	Glutathione Peroxidase;
ELISA	enzyme-linked immunosorbent assay;
CCK-8	Cell Counting Kit-8;
OD	optical density.
