# Supplementary material for: Lipid Dysregulation Induced by Gasoline and Diesel Exhaust Exposure and the Interaction with Age
Source: Toxics. 2024 Apr 19;12(4):303. doi: 10.3390/toxics12040303 (PMC11054039; doi:10.3390/toxics12040303)
Supplement: Supplementary file 1 [file toxics-12-00303-s001.zip › toxics-2953396-supplementary.pdf]

Supplemental Materials

Supplemental Figure

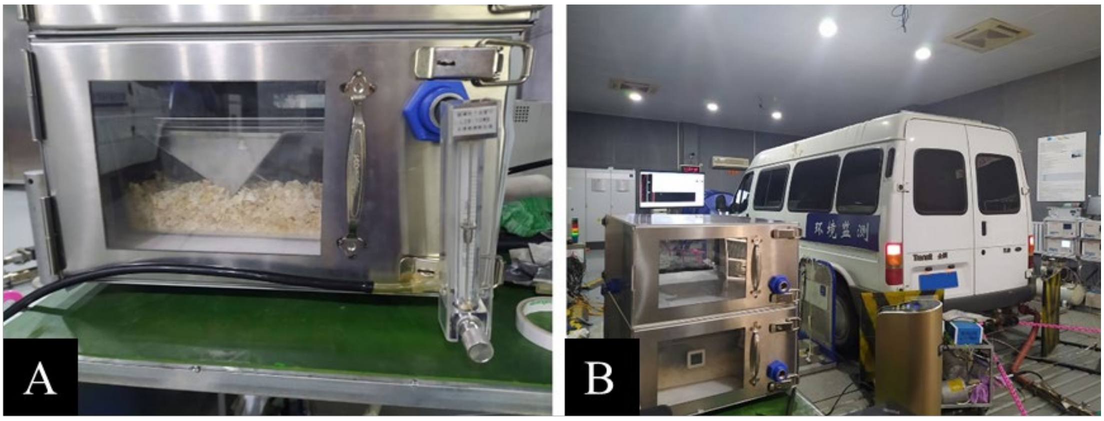

Fig. S1 (A) The exposure chamber; (B) The exposure process

Supplemental Table

Table S1. Typical quantity-size distribution of $PM_{2.5}$

Sampling location	Particle size of $PM_{2.5}$ (nm)	15	30	70	120	200	320	500	800	1700	2400
Wujinglu Tunnel	Mass percentage of $PM_{2.5}$ (%)	0.01	0.26	2.37	4.64	10.19	20.02	27.37	13.75	14.63	6.76
Teda Street	Mass percentage of $PM_{2.5}$ (%)	0.02	0.12	0.42	1.62	5.85	13.88	18.79	23.84	20.22	15.24

Supplemental Text

Text S1 Calculation of the dose.

$$E = C \times B \times t$$

E: Inhalation exposure of pollutant, μg;

C: The concentration of pollutant, μg/m^3;

B: respiratory rate, m^3/d. For human beings, the value equals to 10.8 m^3/d;

t: exposure time, d.
